# Placenta Prævia

**Published:** 1879-01

**Authors:** 


### EXCERPTA.

PLACENTA PRÆVIA.—Dr. Sutton supposes a case, which may happen to any general practitioner, and which has happened to himself. The physician is called to attend an obstetrical case several miles in the country. He has no reason to anticipate, but on arriving at his patient's bedside, finds her as follows: "The woman has been flooding; she is pale; the pulse is small and feeble; the bed is saturated with blood; and, on inquiry, it is probably found that this patient has had occasional attacks of flooding fer several weeks; but as the woman was strong and vigorous, and her hemorrhages lasted only a few minutes, no danger was apprehended. On making an examination, the vagina is found filled with clotted blood; the os is probably dilated to about the size of a quarter or half of a dollar, and, immediately above it, is felt the spongy mass of the placenta revealing beyond all doubt the alarming fact that he has a case of placenta previa. From the loss of blood that has already taken place, and the danger which is so evident, consultation is desired—for it is his wish that another physician should bear a portion of the responsibility in the treatment of a case attended with so much danger and anxiety. But this patient is five or ten miles in the country; the roads are bad, and hours would elapse before a physician could arrive, or Barnes' or Molesworth's dilators be procured." Shall we try the tampon until the uterus is sufficiently dilated to enable us to turn and deliver? Dr. Sutton says his "experience is not favorahle to the tampon." Complete detachment of the placenta, as recommended by Simpson, insures the death of the child, though the condition of the mother may be so urgent in some cases as to make this desirable means of saving her life. But suppose we have decided not to resort to this plan. When we come to the method which Dr. Sutton urges, he says: "We have, then, a modification of the old plan of forcible dilation of the os—using the hand, and, at the same time, a roll of cloths, pressed against the bleeding utero-placental vessels as an internal tampon, while we separate a portion of the placenta, and, at the same time, produce as rapidly as practicable mechanical expansion of the cervix to favor the expulsion of the child, or enable us to effect podalic version and delivery as soon as possible. By this means we have the tampon and forcible dilation, while with the vagina tampon alone we attempt to arrest the hemorrhage, depending upon nature to produce the dilation." He thinks that with "cautious and judicious management we may accomplish with the hand almost all that we can with the rubber dilators. It can be made conical in form, and can be used as a dilating wedge, imitating to some extent the mechanical expansion of the cervix by the 'bag of waters.' Its motions are always under intelligent direction; we know exactly what we are doing."

If the os should continue rigid and does not dilate, Dr. Sutton thinks there would be strong reason for resorting to *free incisions of the cervix.*

In support of the views presented, a number of cases are related, in which the lives of mother and child were saved by methods essentially of this kind: dilation of the os with the fingers; partial separation of the placenta; the introduction of the conical muslin tampon; the introduction of the hand and rupturing the membranes and turning.—*Obstetric Gazette.*

